# High Pretreatment Mean Corpuscular Volume Can Predict Worse Prognosis in Patients With Esophageal Squamous Cell Carcinoma who Have Undergone Curative Esophagectomy: A Retrospective Multicenter Cohort Study

**DOI:** 10.1097/AS9.0000000000000165

**Published:** 2022-05-02

**Authors:** Naoya Yoshida, Ken Sasaki, Kengo Kanetaka, Yasue Kimura, Tomotaka Shibata, Makoto Ikenoue, Yuichiro Nakashima, Noriaki Sadanaga, Kojiro Eto, Yusuke Tsuruda, Shinichiro Kobayashi, Tomonori Nakanoko, Kosuke Suzuki, Shinsuke Takeno, Manabu Yamamoto, Masaru Morita, Yasushi Toh, Hideo Baba

**Affiliations:** From the * Department of Gastroenterological Surgery, Graduate School of Medical Sciences, Kumamoto University, Chuoku, Kumamoto, Japan; † Department of Digestive Surgery, Breast and Thyroid Surgery, Graduate School of Medical and Dental Sciences, Kagoshima University, Kagoshima-shi, Kagoshima, Japan; ‡ Department of Surgery, Nagasaki University Graduate School of Biomedical Sciences, Nagasaki, Japan; § Department of Surgery and Science, Graduate School of Medical Sciences, Kyushu University, Higashi-ku, Fukuoka, Japan; ∥ Department of Gastroenterological and Pediatric Surgery, Oita University, Hasama, Oita, Japan; ¶ Division of Gastrointestinal-Endocrine-Pediatric Surgery, Department of Surgery, Faculty of Medicine, University of Miyazaki, Kiyotake, Miyazaki, Japan; # Department of Gastroenterological Surgery, National Hospital Organization, Kyushu Cancer Center, Miniami-ku, Fukuoka, Japan; ** Department of Surgery, Saiseikai Fukuoka General Hospital, Chuo-ku, Fukuoka, Japan.

**Keywords:** esophageal squamous cell carcinoma, esophagectomy, mean corpuscular volume, prognosis

## Abstract

**Objective:**

To establish the prognostic value of mean corpuscular volume (MCV) in patients with esophageal squamous cell carcinoma (ESCC) who have undergone esophagectomy.

**Background:**

The MCV increases in patients with high alcohol and tobacco consumption. Such a lifestyle can be a risk factor for malnutrition, comorbidities related to those habits, and multiple primary malignancies, which may be associated with frequent postoperative morbidity and poor prognosis.

**Methods:**

This study included 1673 patients with ESCC who underwent curative esophagectomy at eight institutes between April 2005 and November 2020. Patients were divided into normal and high MCV groups according to the standard value of their pretreatment MCV. Clinical background, short-term outcomes, and prognosis were retrospectively compared between the groups.

**Results:**

Overall, 26.9% of patients had a high MCV, which was significantly associated with male sex, habitual smoking and drinking, multiple primary malignancies, and malnutrition, as estimated by the body mass index, hemoglobin and serum albumin values, and the Geriatric Nutritional Risk Index. Postoperative respiratory morbidity (*P* = 0.0075) frequently occurred in the high MCV group. A high MCV was an independent prognostic factor for worse overall survival (hazard ratio, 1.27; 95% confidence interval, 1.049–1.533; *P* = 0.014) and relapse-free survival (hazard ratio, 1.23; 95% confidence interval, 1.047–1.455; *P* = 0.012).

**Conclusions:**

A high MCV correlates with habitual drinking and smoking, malnutrition, and multiple primary malignancies and could be a surrogate marker of worse short-term and long-term outcomes in patients with ESCC who undergo esophagectomy.

## Abstract 2

Mini-Abstract: This multicenter cohort study elucidated that a high pretreatment mean corpuscular volume correlated with habitual drinking and smoking, malnutrition, and multiple primary malignancies, and could be a surrogate marker of worse short-term and long-term outcomes in patients with esophageal squamous cell carcinoma who have undergone curative esophagectomy.

## INTRODUCTION

Esophageal squamous cell carcinoma (ESCC) is the most common histological type of esophageal cancer worldwide. Notably, it frequently occurs in Eastern Asia, including Japan.^1,2^ Alcohol and tobacco consumption and male sex are the representative risk factors for the incidence of ESCC.^2^ Despite advances in recent multidisciplinary treatments, the prognosis of ESCC remains insufficient, and it is the sixth most common cause of cancer-related death in the world.^1^ In Japan, although the 5-year survival rate after surgery has improved to approximately 60%, that for patients in advanced stages remains low (cStage III, IVa, and IVb: 45%, 30%, and 17%, respectively).^3^ Thus, further advances in agents and treatment strategies are required.

Prognostic markers are useful for estimating the treatment outcomes and determining treatment strategies that may contribute to further improvement of the treatment outcomes. Especially, those prognostic markers that are based on laboratory data are clinically useful because they can be easily obtained without any physical or economic burden. The neutrophil-to-lymphocyte ratio,^4^ lymphocyte-to-monocyte ratio,^5^ platelet-to-lymphocyte ratio,^6^ adapted systemic inflammation score,^7^ Glasgow prognostic score,^8^ and red blood cell distribution width^9^ have been previously suggested to be useful in estimating prognosis after esophagectomy for esophageal cancer. Moreover, several nutritional markers, such as the Controlling Nutritional Status (CONUT) score^10^ and prognostic nutritional index (PNI),^11^ are also useful.

The mean corpuscular volume (MCV) is one such laboratory value commonly examined before esophagectomy. It indicates the average volume of red blood cells and is used to estimate the pathogenesis of anemia. The MCV increases with vitamin B12 and folic acid deficiency, which is often observed in patients with habitual drinking.^12^ A high MCV also epidemiologically correlates with habitual smoking,^13^ which might be a predictor of ESCC incidence.^14^ A lifestyle of high alcohol and tobacco consumption can be a risk factor for malnutrition, comorbidities related to those habits, and multiple primary malignancies, which can be risk factors for postoperative morbidity and poor prognosis.^10,15–17^ Thus, we hypothesized that the MCV could be a prognostic marker in patients with ESCC who have undergone esophagectomy. We previously published the first report of a single-institute cohort study on the association of high MCV with poor prognosis after esophagectomy.^18^ Therefore, to validate and further establish the prognostic value of the MCV, we conducted a multicenter cohort study described herein.

## METHODS

### Ethical Approval and Consent to Participate

This study was conducted in conformity to the 1975 Declaration of Helsinki. The study procedures were performed after acquiring approval from the ethical committee of each participating institute with a waiver of written informed consent [registry numbers: 2191 (Kumamoto University), 210010 (Kagoshima University), 20151713 (Nagasaki University), 2021-109 (Kyushu University), 2066 (Oita University), 0-0919 (Miyazaki University), 2021-12 (Kyushu Cancer Center), and 2021-3-1 (Saiseikai Fukuoka General Hospital)].

### Study Design and Patients

This multicenter cohort study included 1813 patients with ESCC who underwent curative esophagectomy at 8 hospitals in the Kyushu region of Japan between April 2005 and November 2020 (see Supplemental Figure 1, http://links.lww.com/AOSO/A122, which shows a study flow chart). Of these, 113 patients with insufficient data on patient characteristics, tumor characteristics, surgical background, and prognosis were excluded. Additionally, 27 patients with low MCV were excluded because this was a rather small sample size to analyze. Consequently, 1673 patients were included. Pretreatment MCV was examined in all the patients. The patients were categorized into two groups: normal (83–99 fL) and high (>99 fL) groups, according to the standard MCV value.^19^

### Data Collection

Data were collected from the clinical database of each institution. Associations between the pretreatment MCV and clinical background, short-term outcome, and prognosis were retrospectively examined. Pretreatment clinical stage (cStage) was classified based on the Union for International Cancer Control TNM staging version 8.^20^ cStage IVB was included in this study only when it was due to M1 lymph nodes (LNs), which corresponded to regional LNs in the Japanese Classification of Esophageal Cancer (ie, supraclavicular LNs).^21^

### Institutional Authorization

All 8 hospitals that participated were Authorized Institutes for Board-Certified Esophageal Surgeons (AIBCES) certified by the Japan Esophageal Society (JES). The provisions for certification are available elsewhere.^22^ The AIBCES need to meet eight criteria to be certified. Clinically, ≥100 treated patients and ≥50 surgeries for esophageal disease per 5 years and full-time employment are required of board-certified esophageal surgeons. A cohort study using 4897 patients with esophageal cancer registered in the Japan National Database of Hospital-based Cancer Registries, suggested that survival outcomes after esophagectomy at AIBCES were significantly better than those at non-AIBCES.^23^

### Esophagectomy

Several types of esophagectomy were included in this study if each esophagectomy was curatively performed. Curative esophagectomy was defined as surgery with surgical R0 and R1 resections. R2 resection, which indicates residual macroscopically visible cancer during esophagectomy, was excluded.

### Definition of Morbidity

Postoperative morbidity and severe morbidity were defined as a complication of the Clavien-Dindo classification (CDc) grade ≥II and ≥IIIb, respectively.^24^ Definitions of pneumonia, respiratory morbidity, cardiovascular morbidity, surgical site infection, and leakage are available elsewhere.^25^

### Definition of Prognostic Outcome

Overall survival (OS) was defined as the interval from the date of surgery to death. Relapse-free survival (RFS) was defined as the interval from the date of surgery to recurrence and death.

### Statistical Analysis

The Mann–Whitney U test was employed for statistical comparison between the unpaired samples. The chi-square test was employed for statistical comparison between the groups. Analysis for the survival time distribution and significant differences on OS and RFS was conducted using the Kaplan–Meier method and log-rank test, respectively. Investigation for independent risk factors and the hazard ratio (HR) was performed using a multivariate Cox proportional hazards model. Univariate analysis was performed to examine the effect of the following factors: age (for a 10-year increase); male sex [vs female sex]; body mass index (BMI) <18.5 (vs ≥18.5 kg/m^2^); Brinkman index [number of cigarettes per day × smoking duration (year), for an increase of 100]; performance status (PS) 0 (vs ≥1); the American Society of Anesthesiologists physical status 1 and 2 (vs 3); cStage II, III, IVA, and IVB (vs I); number of dissection fields 0 and 1 (vs 2 and 3); operative time (for a 60-minute increase); bleeding (for a 100-g increase); severe morbidity (vs no); and MCV high (vs normal). Factors that showed a probability of *P* <0.10 were subjected to the final analysis. An independent risk factor was considered appropriate at a *P* value <0.05. Ultimately, modification of the effect of MCV on OS by other parameters was examined. The study period was included in the analysis to minimize historical bias regarding perioperative cancer treatment. The study period was divided into 3 phases: before the JCOG9907 study (2005−2011), the transitional period (2012−2013), and after generalization (2014−2020), because the standard treatment for locally advanced ESCC in Japan has been modified from surgery plus adjuvant chemotherapy to neoadjuvant chemotherapy plus subsequent surgery since 2012 (JCOG9907).^26^ JMP version 13.1 software (SAS Institute, Cary, NC) and StatView software package (version 5.0; Abacus Concepts, Inc., Berkeley, CA) were used for statistical analysis.

## RESULTS

### Patients’ Clinical Features

Of 1673 patients, 450 (26.9%) had a high pretreatment MCV (Table 1). A high MCV significantly correlated with younger age (*P* < 0.0001), male sex (*P* < 0.0001), habitual smoking (*P* < 0.0001), habitual drinking (*P* < 0.0001), as well as with frequent synchronous or metachronous multiple primary malignancies (*P* = 0.0015). Moreover, a high MCV was significantly associated with lower BMI (*P* < 0.0001), hemoglobin level (*P* < 0.0001), serum albumin value (*P* = 0.027), and Geriatric Nutritional Risk Index (GNRI) (*P* < 0.0001), suggesting that MCV could be a surrogate marker of malnutrition (Supplemental Table 2, http://links.lww.com/AOSO/A122, shows the associations).

**TABLE 1.** Pretreatment MCV and Patients’ Characteristics

Clinical and Epidemiological Features	Total N	Pretreatment MCV		*P*
		Normal	High	
All cases	1673	1223	450	
Mean age ± SD (years)	65.9 ± 8.5	66.4 ± 8.6	64.3 ± 8.2	<0.0001
Male sex	1406 (84%)	1002 (82%)	404 (90%)	<0.0001
Mean body mass index ± SD (kg/m^2^)	21.6 ± 3.2	21.8 ± 3.2	20.9 ± 2.9	<0.0001
Body mass index (kg/m^2^)				<0.0001
<18.5	275 (16%)	176 (14%)	99 (22%)	
18.5–25.0	1181 (71%)	863 (71%)	318 (71%)	
>25.0	217 (13%)	184 (15%)	33 (7%)	
Brinkman index ± SD*	740 ± 580	710 ± 580	820 ± 570	0.0005
Habitual tobacco use, yes	1377 (82%)	976 (80%)	401 (89%)	<0.0001
Habitual alcohol use, yes†	1446 (88%)	1025 (85%)	421 (95%)	<0.0001
Performance status				0.12
0	1452 (87%)	1052 (86%)	400 (89%)	
≥1	221 (13%)	171 (14%)	50 (11%)	
American Society of Anesthesiologists physical status				0.68
1 and 2	1585 (95%)	1157 (95%)	428 (95%)	
3	88 (5%)	66 (5%)	22 (5%)	
Synchronous or metachronous multiple primary malignancy, yes	490 (29%)	332 (27%)	158 (35%)	0.0015

*The Brinkman index was calculated as follows: number of cigarettes per day × smoking duration (year).

†31 missing data existed.

### Tumor and Treatment Characteristics

The tumor location was statistically different between the groups (*P* = 0.022). However, other characteristics, such as the cStage, preoperative treatment, surgical procedure, and number of LN dissection fields, were equivalent between the groups (Table 2).

**TABLE 2.** Pretreatment MCV and Tumor and Treatment Characteristics

Tumor and Surgical Characteristics	Total N	Pretreatment MCV		*P*
		Normal	High	
All cases	1673	1223	450	
Location of the tumor				0.022
Ce	60 (4%)	36 (3%)	24 (5%)	
Ut	234 (14%)	173 (14%)	61 (14%)	
Mt	858 (51%)	617 (50%)	241 (54%)	
Lt	475 (28%)	357 (29%)	118 (26%)	
Ae	46 (3%)	40 (3%)	6 (1%)	
Clinical stage				0.42
I	612 (37%)	437 (36%)	175 (39%)	
II	349 (21%)	250 (20%)	99 (22%)	
III	510 (30%)	388 (32%)	122 (27%)	
IVA	117 (7%)	84 (7%)	33 (7%)	
IVB*	85 (5%)	64 (5%)	21 (5%)	
Preoperative treatment, yes	857 (51%)	636 (52%)	221 (49%)	0.29
Surgical procedure				0.88
Three-incision esophagectomy	1534 (92%)	1125 (92%)	409 (91%)	
Ivor Lewis	14 (1%)	11 (1%)	3 (1%)	
Transhiatal esophagectomy	47 (3%)	34 (3%)	13 (3%)	
Pharyngolaryngoesophagectomy	50 (3%)	34 (3%)	16 (4%)	
Mediastinoscopic esophagectomy	23 (1%)	15 (1%)	8 (2%)	
Others	5 (0.3%)	4 (0.3%)	1 (0.2%)	
Thoracic procedure				0.99
Minimally invasive†	1219 (73%)	891 (73%)	328 (73%)	
Open	454 (27%)	332 (27%)	122 (27%)	
Abdominal procedure				0.60
Laparoscopic	778 (47%)	564 (46%)	214 (48%)	
Open	895 (53%)	659 (54%)	236 (52%)	
No. dissection fields				0.25
0 and 1	97 (6%)	66 (5%)	31 (7%)	
2 and 3	1576 (94%)	1157 (95%)	419 (93%)	

*cStage IVB included only cancers with clinical M1 lymph nodes according to the Union for International Cancer Control TNM staging corresponding to regional lymph nodes in the Japanese Classification of Esophageal Cancer.

†Minimally invasive procedures in the thorax included thoracoscopic, mediastinoscopic, and transhiatal surgeries.

Ae indicates abdominal esophagus; Ce, cervical esophagus; Lt, lower thoracic esophagus; Mt, middle thoracic esophagus; Ut, upper thoracic esophagus.

### Short-Term Outcomes After Surgery

Table 3 presents short-term outcomes after surgery. The mean operative time was significantly longer in the high MCV group than in the normal MCV group, although the difference was only 15 minutes. Regarding postoperative morbidity, pneumonia (*P* = 0.029) and respiratory morbidity (*P* = 0.0075) frequently occurred in the high MCV group. Results of the logistic regression analysis indicated that a high MCV was an independent risk factor for postoperative respiratory morbidity [HR, 1.46; 95% confidence interval (CI), 1.094–1.956; *P* = 0.010], along with an increased Brinkman index, and open procedure in the thorax (Supplemental Table 3, http://links.lww.com/AOSO/A122, shows detailed results of the analysis).

**TABLE 3.** Pretreatment MCV and Short-Term Outcomes of surgery

Surgical Data and Morbidity	Total N	Pretreatment MCV		*P*
		Normal	High	
All cases	1673	1223	450	
Mean operative time ± SD (min)	560 ± 130	550 ± 130	570 ± 130	0.03
Mean bleeding ± SD (g)	430 ± 490	430 ± 480	430 ± 500	0.85
Postoperative morbidity (CDc ≥II)	679 (41%)	480 (39%)	199 (44%)	0.066
Postoperative severe morbidity (CDc ≥IIIb)	152 (9%)	103 (8%)	49 (11%)	0.12
Pneumonia	235 (14%)	158 (13%)	77 (17%)	0.029
Pulmonary morbidity	265 (16%)	176 (14%)	89 (20%)	0.0075
Cardiovascular morbidity	66 (4%)	49 (4%)	17 (4%)	0.83
Surgical site infection	430 (26%)	303 (25%)	127 (28%)	0.15
Leak	294 (18%)	209 (17%)	85 (19%)	0.39

### Prognosis After Surgery

**FIGURE 1.**
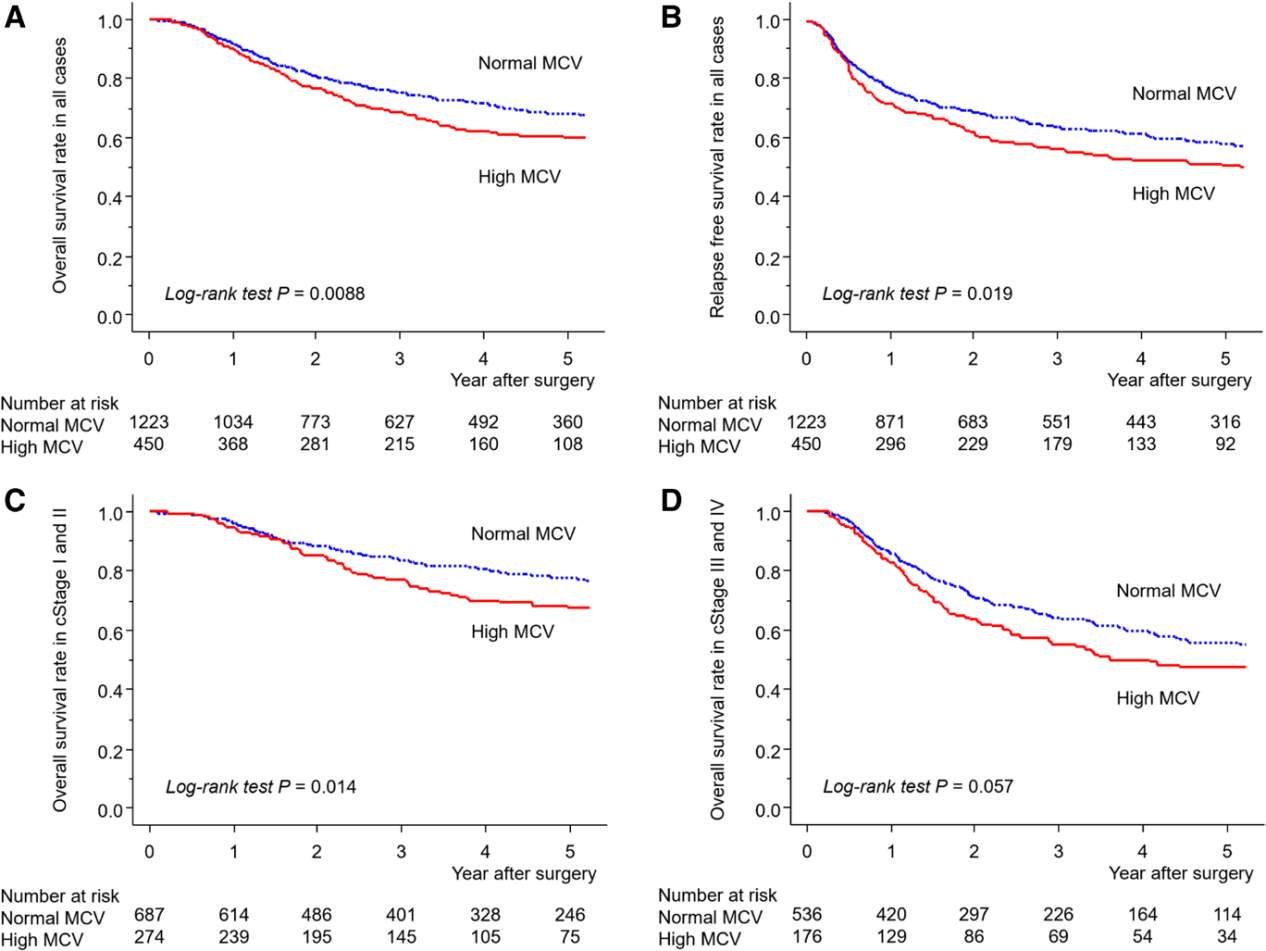
Kaplan–Meier curves of os (A), RFS (B) in all patients, and overall survival in clinical stages I and II (C) and III and IV (D) esophageal squamous cell carcinoma in accordance with the status of the pretreatment MCV.

**FIGURE 2.**
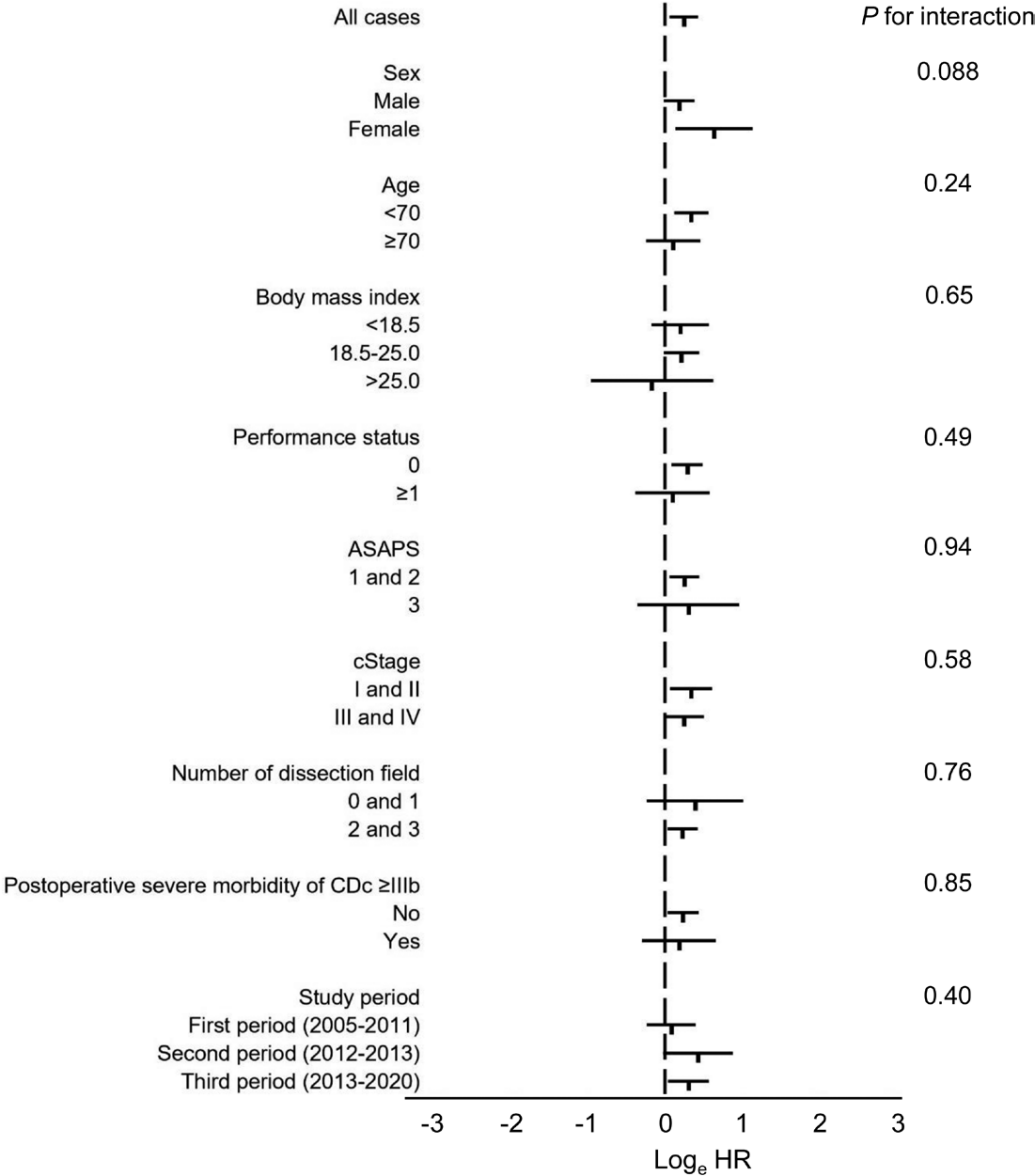
Relationship between the MCV and OS in various strata: log (HRs) plots showing the 95% confidence interval for the overall survival rate related to a high mean corpuscular volume. ASAPS indicates American Society of Anesthesiologists physical status.

Figure 1 presents the Kaplan–Meier curves of OS and RFS between the groups. A high MCV was significantly associated with worse OS (*P* = 0.0088) and RFS (*P* = 0.019) in all the patients (Figs. 1A, B). Further analysis suggested that the OS in cStages I and II was significantly worse in the high MCV group than in the normal group (*P* = 0.014) (Fig. 1C). Moreover, a high MCV exhibited a trend toward a worse OS in cStages III and IV, although it was not statistically significant (*P* = 0.057) (Fig. 1D). Results of the Cox regression analysis suggested that a high MCV was an independent prognostic factor for worse OS (HR, 1.27; 95% CI, 1.049–1.533; *P* = 0.014), along with advanced age, low BMI, worse American Society of Anesthesiologists physical status, advanced cStage, small number of dissection fields, higher blood loss, and severe postoperative morbidity of CDc ≥IIIb (Table 4), as well as for worse RFS (HR, 1.23; 95% CI, 1.047–1.455; *P* = 0.012) (Supplemental Table 4, http://links.lww.com/AOSO/A122, shows detailed results of the Cox regression analysis). Regarding the influence of MCV on OS, modification by other clinical parameters was not observed (Fig. 2).

**TABLE 4.** Results of Cox Regression Analysis of OS

	Univariate Analysis		Multivariate Analysis	
Characteristics	HR (95% CI)	*P*	HR (95% CI)	*P*
Age (for a 10-year increase)	1.14 (1.027–1.264)	0.013	1.16 (1.037–1.291)	0.0090
Male sex (vs female sex)	1.08 (0.849–1.373)	0.53		
Body mass index <18.5 (vs ≥18.5) (kg/m^2^)	1.81 (1.474–2.228)	<0.0001	1.41 (1.132–1.747)	0.0020
Brinkman index (for an increase of 100)	1.002 (0.987–1.017)	0.80		
Performance status 0 (vs ≥1)	0.45 (0.252–0.791)	0.0057	0.83 (0.643–1.062)	0.14
American Society of Anesthesiologists physical statuses 1 and 2 (vs 3)	0.46 (0.335–0.621)	<0.0001	0.58 (0.414–0.817)	0.0018
Clinical stage II (vs stage I)	1.89 (1.461–2.449)	<0.0001	1.87 (1.436–2.425)	<0.0001
Clinical stage III (vs stage I)	2.50 (1.989–3.153)	<0.0001	2.44 (1.926–3.088)	<0.0001
Clinical stage IVA (vs stage I)	4.10 (3.024–5.559)	<0.0001	3.46 (2.524–4.736)	<0.0001
Clinical stage IVB* (vs stage I)	3.87 (2.708–5.533)	<0.0001	4.03 (2.807–5.797)	<0.0001
No. dissection fields, 0 and 1 (vs 2 and 3)	1.71 (1.247–2.348)	0.0009	1.51 (1.087–2.087)	0.014
Operative time (for a 60-minute increase)	1.04 (0.998–1.080)	0.064	1.01 (0.968–1.052)	0.67
Bleeding (for a 100-g increase)	1.03 (1.019–1.041)	<0.0001	1.02 (1.006–1.032)	0.0040
Severe morbidity of CDc ≥IIIb (vs no)	1.87 (1.456–2.389)	<0.0001	1.73 (1.339–2.233)	<0.0001
MCV high (vs normal)	1.28 (1.063–1.538)	0.0090	1.27 (1.049–1.533)	0.014

*cStage IVB included only cancers with clinical M1 lymph nodes according to the Union for International Cancer Control TNM staging corresponding to regional lymph nodes in the Japanese Classification of Esophageal Cancer.

## DISCUSSION

Alcohol and tobacco use are 2 representative risk factors for the incidence of ESCC, and MCV epidemiologically reflects alcohol and tobacco consumption.^12,13,18^ The average MCVs in male and female patients in this study were 96.4 and 94.2 fL, respectively, which were considerably higher than those in the general healthy population in Japan (91.0 and 90.7 fL, respectively).^19^ Consequently, as many as 27% of the patients with ESCC had a high pretreatment MCV. High alcohol and tobacco consumption may have several adverse effects on surgical and survival outcomes, such as malnutrition, comorbidity related to those habits, and multiple primary malignancies.^10,15,16^ In the present multicenter cohort study, several interesting results were obtained that confirm our hypothesis: (1) high pretreatment MCV significantly correlated with habitual drinking and smoking, a high Brinkman index and male sex, which are risk factors for ESCC; (2) high MCV correlated with malnutrition, as estimated by the BMI, hemoglobin level, serum albumin value, and GNRI; (3) high MCV significantly correlated with synchronous or metachronous malignancies other than ESCC; (4) high MCV was a significant risk factor for postoperative pneumonia and respiratory morbidity; and (5) high MCV was an independent risk factor for poor OS and RFS.

In this study, we analyzed the MCV values before any treatment administration. The preoperative value was often used to investigate the postesophagectomy prognostic value of the hematologic parameters. However, preoperative treatments are commonly administered for locally advanced ESCC, which could affect the MCV value and complicate the elucidation of the background that may worsen prognosis in patients with a high MCV. Therefore, we used the pretreatment MCV to clarify the correlation between a high MCV and poor prognosis.

A high MCV was associated with habitual drinking and smoking. Previous studies have suggested that drinking and smoking could be risk factors for postoperative morbidity after esophagectomy.^27,28^ Impairment of liver and respiratory functions, respiratory comorbidity, and cardiovascular comorbidity related to alcohol and tobacco can increase postesophagectomy morbidities.^15^ In particular, smoking promotes proinflammatory cytokine release, chronic airway inflammation, and mucus hypersecretion,^29^ which could contribute to the increase in respiratory morbidity. Additionally, these disadvantageous backgrounds also increase deaths due to diseases other than esophageal cancer, which can also worsen survival outcomes.

A high MCV could be a surrogate marker of poor nutritional status, as inferred from BMI, hemoglobin and serum albumin values, and GNRI. Malnutrition delays tissue repair and reduces host immune function,^30^ which can increase postoperative morbidity. A decrease in activities of daily living (ADL) in patients with malnourishment and sarcopenia can delay postoperative recovery and increase respiratory morbidity after esophagectomy due to emaciation of breathing muscles and difficulty in sputum evacuation.^31,32^ The GNRI has been suggested to predict postoperative morbidity and prognosis after esophagectomy.^33,34^ Additionally, several studies have suggested that malnutrition assessed by the CONUT score and PNI may increase postoperative morbidities after esophagectomy.^25,35^ Studies on the association between malnutrition and postesophagectomy morbidity may explain a probable mechanism of increased morbidity in patients with a high MCV.

A high MCV correlated with frequent multiple primary malignancies other than ESCC, possibly due to habitual drinking and smoking.^36^ Multiple primary malignancies themselves can be a potent reason for increased death.^16^ Moreover, a history of surgery for metachronous malignancy can complicate esophagectomy due to adhesion. A previous history of gastrointestinal surgery may complicate the reconstruction. The presence of simultaneous malignancy can also complicate surgery because of concurrent resection. These factors can increase postoperative morbidity and further worsen the prognosis after esophagectomy.

Postoperative morbidities worsen prognosis after the surgical treatment of cancer. Notably, respiratory, infectious, and severe morbidities were suggested to deteriorate prognosis after esophagectomy for esophageal cancer.^37–39^ Persistent inflammation affects cytokine reactions and antitumor immunity, which can promote residual cancer cell growth.^39^ Prolonged recovery after esophagectomy can be a reason for the loss of the appropriate timing of adjuvant treatment. Deterioration of PS and ADL after severe morbidity can be a reason for avoiding invasive treatment for recurrence. Besides, a low PS and ADL can also be risk factors of death due to impaired physical condition, such as aspiration pneumonia and traumatic injury.^18,40^ Thus, the increased postoperative morbidity in patients with a high MCV may worsen the prognosis after esophagectomy.

**FIGURE 3.**
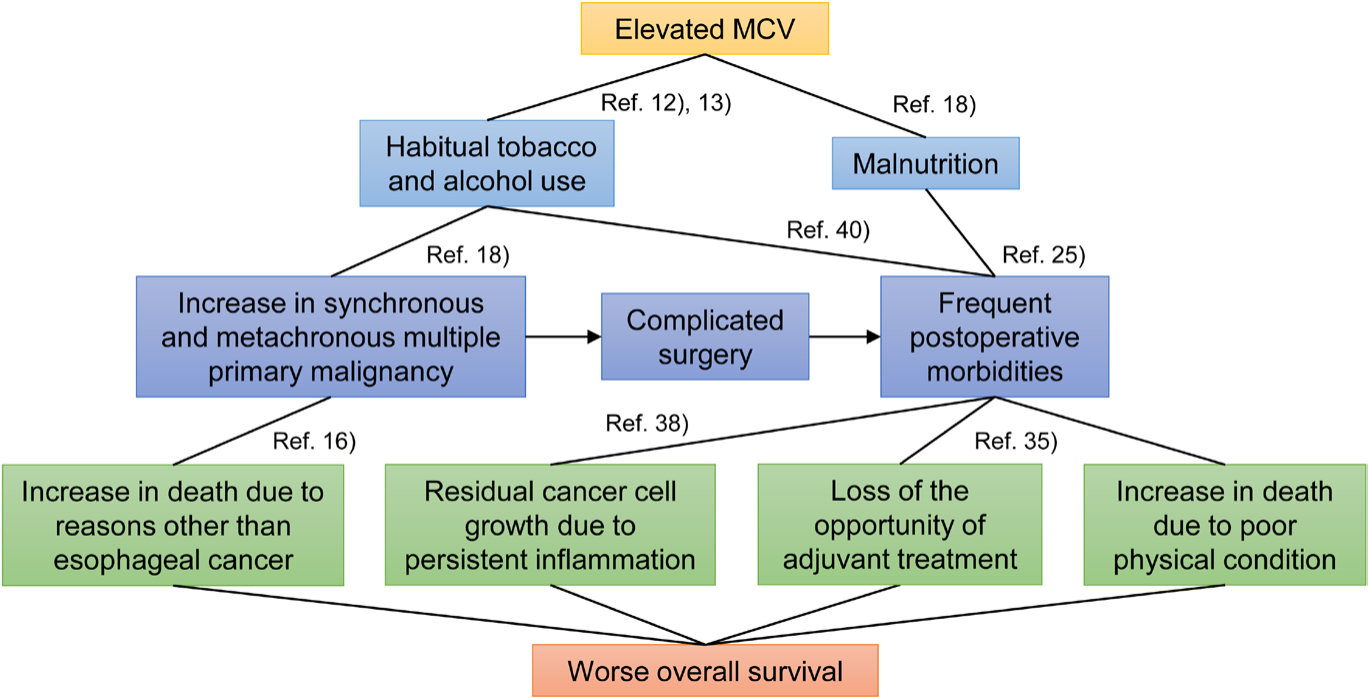
Summary of the association of an elevated MCV with poor prognosis.

Based on the current results, a high pretreatment MCV reflects past drinking and smoking, malnutrition, and an increase in multiple primary cancers, which can increase postoperative morbidity and ultimately worsen prognosis (Fig. 3). Therefore, for ESCC patients with a high pretreatment MCV for whom esophagectomy is planned, clarification of the underlying risks and prophylaxes for postoperative morbidity are considerably important. Preoperative smoking cessation,^41^ nutritional support,^42^ respiratory rehabilitation,^43^ and oral hygiene^44^ may help improve surgical outcomes. Minimally invasive esophagectomy has been reported to have fewer postoperative morbidities irrespective of the type of preoperative treatment.^15^ Thus, performing minimally invasive surgery is one of the possible options.^45^ Preoperative prediction of postesophagectomy morbidities is also important in preventing and minimizing them. Objective predictors, such as exhaled carbon monoxide,^46^ left-sided esophagus on computed tomography (CT),^47^ and asymptomatic sputum in the respiratory tract on CT^32^ may be useful in predicting morbidity.

This is the largest cohort study to explore the correlation between hematologic parameters and prognosis after esophagectomy for ESCC. All facilities participating in this study were AIBCESs certified by the JES. Hence, sufficient quality of treatment strategy, surgery, and perioperative management were maintained among the institutions. Nevertheless, this study has several limitations. Because of the retrospective, multicenter nature of the studies, there might have been inter-institutional biases regarding the accuracy of preoperative clinical staging, esophagectomy volume, surgical procedure, perioperative management, follow-up schedules, and surgical outcomes, in addition to historical bias regarding perioperative treatment. To minimize historical bias regarding perioperative cancer treatment in JCOG9907, we confirmed that the effect of MCV on OS did not differ among the three study periods. Additionally, several factors that could affect the MCV value, such as vitamin B12 and folic acid deficiency, history of gastrectomy and hypothyroidism, among others, could not be fully screened. Last, the objective area was limited to Japan. Thus, further studies using cohorts in other regions are necessary to establish the universal prognostic value of the MCV in patients with ESCC.

In conclusion, we believe that a high pretreatment MCV correlates with habitual drinking and smoking, malnutrition, and multiple primary malignancies, and could be a surrogate marker of worse short-term and survival outcomes in patients with ESCC who underwent esophagectomy. For patients with a high MCV, it is important to identify the underlying risks and take measures to reduce postoperative morbidity, which may contribute to improving prognosis after esophagectomy.

## Supplementary Material


