# Supplementary material for: High Pretreatment Mean Corpuscular Volume Can Predict Worse Prognosis in Patients With Esophageal Squamous Cell Carcinoma who Have Undergone Curative Esophagectomy: A Retrospective Multicenter Cohort Study
Source: Ann Surg Open. 2022 May 2;3(2):e165. doi: 10.1097/AS9.0000000000000165 (PMC10431247; doi:10.1097/AS9.0000000000000165)
Supplement: Supplementary file 1 [file as9-3-e165-s001.pdf]

SUPPLEMENTAL DIGITAL CONTENT 1

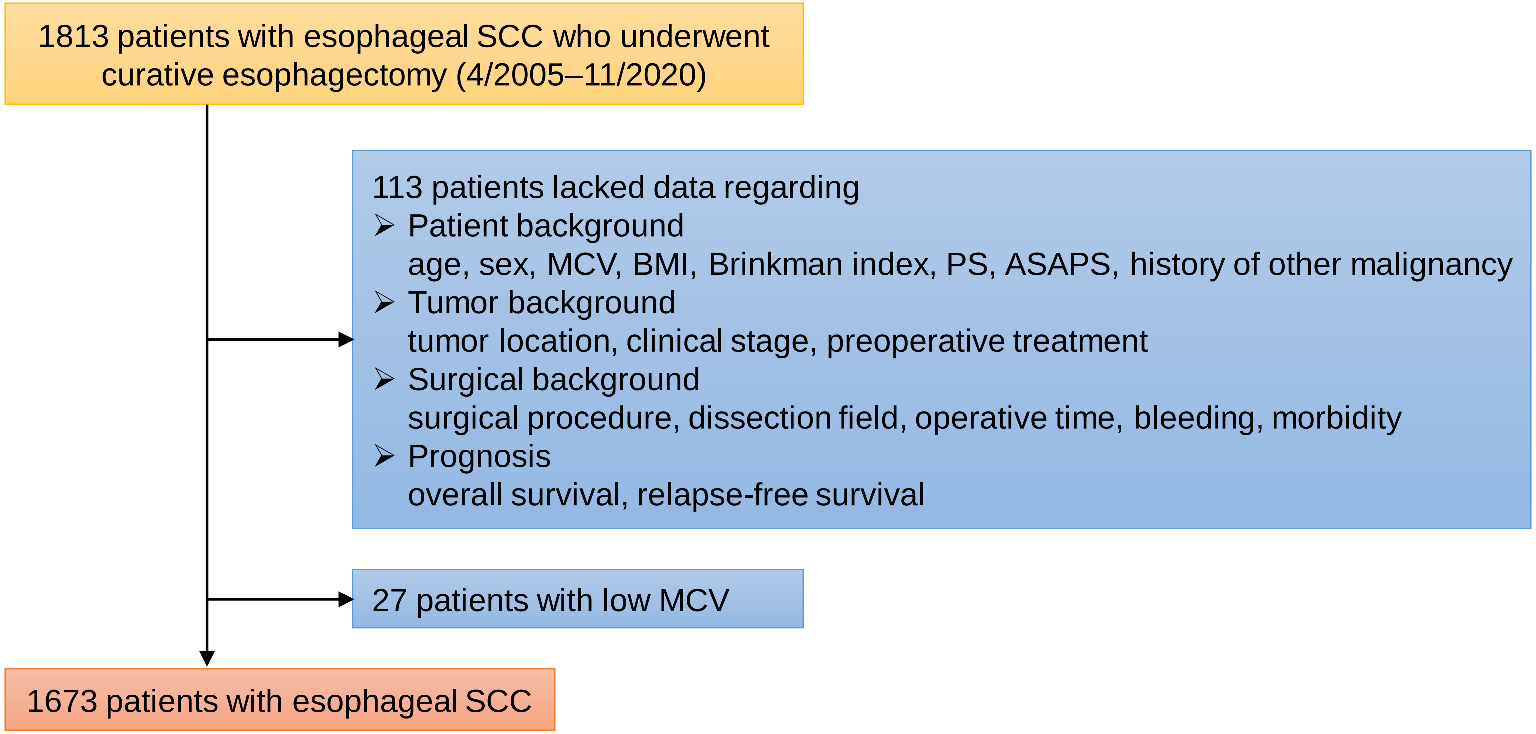

SUPPLEMENTAL DIGITAL CONTENT 2. Associations between the pretreatment mean corpuscular volume, hemoglobin, serum albumin, and Geriatric Nutritional Risk Index

| | | Pretreatment MCV | | *P* |
|---|---|---|---|---|
| Blood data | Total N | Normal | High | |
| All cases | 1673 | 1223 | 450 | |
| Hemoglobin, mean ± SD (g/dL)[#] | 13.6 ± 1.6 | 13.7 ± 1.6 | 13.3 ± 1.5 | <0.0001 |
| ≤13.0 | 568 (34%) | 387 (32%) | 181 (40%) | 0.0008 |
| >13.0 | 1102 (66%) | 835 (68%) | 267 (60%) | |
| Serum albumin, mean ± SD (g/dL)* | 4.1 ± 0.4 | 4.1 ± 0.4 | 4.0 ± 0.4 | 0.027 |
| ≤3.9 | 458 | 306 (25%) | 152 (34%) | 0.0003 |
| >3.9 | 1213 | 916 (75%) | 297 (66%) | |
| Geriatric Nutritional Risk Index, mean ± SD* | 105.7 ± 9.7 | 106.5 ± 9.9 | 103.7 ± 8.9 | <0.0001 |
| <98 | 329 (20%) | 225 (18%) | 104 (23%) | 0.030 |
| ≥98 | 1342 (80%) | 997 (82%) | 345 (77%) | |

*MCV,* mean corpuscular volume; *SD,* standard deviation

[#]Three missing data points exist.

*Two missing data points exist.

SUPPLEMENTAL DIGITAL CONTENT 3. Results of logistic regression analysis of postoperative respiratory morbidity

| | Univariate analysis | | Multivariate analysis | |
|---|---|---|---|---|
| Characteristic | HR (95% CI) | *P* | HR (95% CI) | *P* |
| Age (for a 10-year increase) | 1.16 (0.988–1.353) | 0.071 | 1.18 (0.999–1.396) | 0.052 |
| Male sex (vs. female sex) | 1.66 (1.098–2.495) | 0.016 | 1.42 (0.925–2.186) | 0.11 |
| Body mass index <18.5 (vs. ≥18.5) (kg/$m^2$) | 1.08 (0.764–1.532) | 0.66 | | |
| Brinkman index (for an increase of 100) | 1.04 (1.013–1.056) | 0.0014 | 1.03 (1.003–1.048) | 0.029 |
| Respiratory comorbidity, yes (vs. no) | 1.20 (0.879–1.626) | 0.26 | | |
| Performance status 0 (vs. ≥1) | 0.89 (0.612–1.301) | 0.55 | | |
| American Society of Anesthesiologists physical statuses 1 and 2 (vs. 3) | 0.54 (0.329–0.897) | 0.017 | 0.60 (0.359–1.013) | 0.056 |

| | | | | |
|---|---|---|---|---|
| Clinical stages I and II (vs. stage III and IV) | 0.71 (0.542–0.918) | 0.0095 | 0.84 (0.604–1.163) | 0.29 |
| Preoperative treatment, yes (vs. no) | 1.37 (1.048–1.781) | 0.021 | 1.21 (0.876–1.680) | 0.25 |
| Minimally invasive procedure in the thorax[#] (vs. open) | 0.59 (0.446–0.776) | 0.0002 | 0.65 (0.489–0.874) | 0.0041 |
| Laparoscopic procedure in the abdomen (vs. open) | 0.91 (0.699–1.185) | 0.48 | | |
| Number of dissection fields, 0 and 1 (vs. 2 and 3) | 0.60 (0.305–1.161) | 0.13 | | |
| Operative time (for a 60-min increase) | 1.04 (0.978–1.104) | 0.22 | | |
| Bleeding (for a 100-g increase) | 1.03 (1.005–1.052) | 0.015 | 1.02 (0.998–1.046) | 0.075 |
| Mean corpuscular volume, high (vs. normal) | 1.47 (1.107–1.944) | 0.0077 | 1.46 (1.094–1.956) | 0.010 |

*HR,* hazard ratio; *CI,* confidence interval; *vs.*, versus

[#]Minimally invasive procedures in the thorax included thoracoscopic, mediastinoscopic, and transhiatal surgeries.

SUPPLEMENTAL DIGITAL CONTENT 4. Results of Cox regression analysis of relapse-free survival

| | Univariate analysis | | Multivariate analysis | |
|---|---|---|---|---|
| Characteristics | HR (95% CI) | *P* | HR (95% CI) | *P* |
| Age (for a 10-year increase) | 0.98 (0.900–1.075) | 0.71 | | |
| Male sex (vs. female sex) | 1.004 (0.818–1.233) | 0.97 | | |
| Body mass index <18.5 (vs. ≥18.5) (kg/$m^2$) | 1.52 (1.262–1.833) | <0.0001 | 1.14 (0.945–1.389) | 0.17 |
| Brinkman index (for an increase of 100) | 0.99 (0.982–1.007) | 0.39 | | |
| Performance status 0 (vs. ≥1) | 0.72 (0.584–0.875) | 0.0011 | 0.85 (0.683–1.063) | 0.16 |
| American Society of Anesthesiologists physical statuses 1 and 2 (vs. 3) | 0.63 (0.468–0.841) | 0.0018 | 0.73 (0.533–1.001) | 0.050 |
| Clinical stage II (vs. stage I) | 1.98 (1.585–2.479) | <0.0001 | 1.99 (1.591–2.498) | <0.0001 |

| | | | | |
|---|---|---|---|---|
| Clinical stage III (vs. stage I) | 2.71 (2.219–3.304) | <0.0001 | 2.69 (2.193–3.290) | <0.0001 |
| Clinical stage IVA (vs. stage I) | 4.31 (3.290–5.656) | <0.0001 | 3.80 (2.874–5.021) | <0.0001 |
| Clinical stage IVB[#] (vs. stage I) | 3.88 (2.831–5.314) | <0.0001 | 4.00 (2.911–5.499) | <0.0001 |
| Number of dissection fields, 0 and 1 (vs. 2 and 3) | 1.41 (1.055–1.888) | 0.020 | 1.40 (1.043–1.890) | 0.025 |
| Operative time (for a 60-min increase) | 1.04 (1.008–1.081) | 0.015 | 1.02 (0.983–1.057) | 0.30 |
| Bleeding (for a 100-g increase) | 1.03 (1.022–1.045) | <0.0001 | 1.02 (1.006–1.032) | 0.0054 |
| Severe morbidity of CDc ≥IIIb (vs. no) | 1.48 (1.172–1.873) | 0.0010 | 1.40 (1.100–1.784) | 0.0062 |
| Mean corpuscular volume, high (vs. normal) | 1.22 (1.033–1.430) | 0.019 | 1.23 (1.047–1.455) | 0.012 |

*CDc*, Clavien-Dindo classification; *HR*, hazard ratio; *CI*, confidence interval; *vs.*, versus

[#]cStage IVB included only cancers with clinical M1 lymph nodes according to the Union for International Cancer Control TNM staging corresponding to regional lymph nodes in the Japanese Classification of Esophageal Cancer.
